# Anti‐Tubercular Drug‐Induced Liver Injury: Current Understanding and Emerging Directions

**DOI:** 10.1002/jgh3.70338

**Published:** 2026-01-18

**Authors:** Shubham Prasad, Himanshu Narang, Saurabh Kedia, Vineet Ahuja

**Affiliations:** ^1^ Department of Gastroenterology and Human Nutrition All India Institute of Medical Sciences New Delhi India

**Keywords:** anti‐tubercular treatment, DILI, hepatotoxicity, tuberculosis

## Abstract

Most common adverse effect causing cessation of anti‐tubercular treatment (ATT) is drug‐induced liver injury (DILI) which is unpredictable due to its idiosyncratic nature. ATT is the most common cause of DILI and drug‐induced acute liver failure (ALF) in South East Asia. Spectrum of ATT‐DILI ranges from asymptomatic raised transaminases to acute hepatitis to acute liver failure (ALF). ALF due to ATT has a more aggressive course with up to 70% mortality. Both modifiable and non‐modifiable risk factors are involved. Increasing age, female gender, genetic predisposition, poor nutrition, underlying liver disease, and concomitant viral infections make one prone to ATT‐DILI. Thus, pretreatment evaluation is very important. Diagnosis of ATT‐DILI is challenging due to lack of specific diagnostic tests; rather, it is a diagnosis of exclusion. Mild transient asymptomatic raised transaminases is due to hepatic adaptation and does not require any modification or cessation of ATT. Early detection of clinically significant DILI by frequent monitoring is associated with better prognosis and low mortality. Prompt withdrawal of all the potential hepatotoxic drugs is the key step in the management. Since the benefit of first‐line ATT outweighs the monitored risk, reintroduction is always considered after normalization of raised transaminases. Ideal regimen is sequential reintroduction with incremental dosage of least hepatotoxic drug first, but evidence for this is lacking. Since hepatotoxicity rate is similar across different regimens, reintroduction is individualized based on perceived clinical risk. Future research is needed to identify specific biomarker panel for diagnosing ATT‐DILI.

## 1 Introduction

**FIGURE 1:**
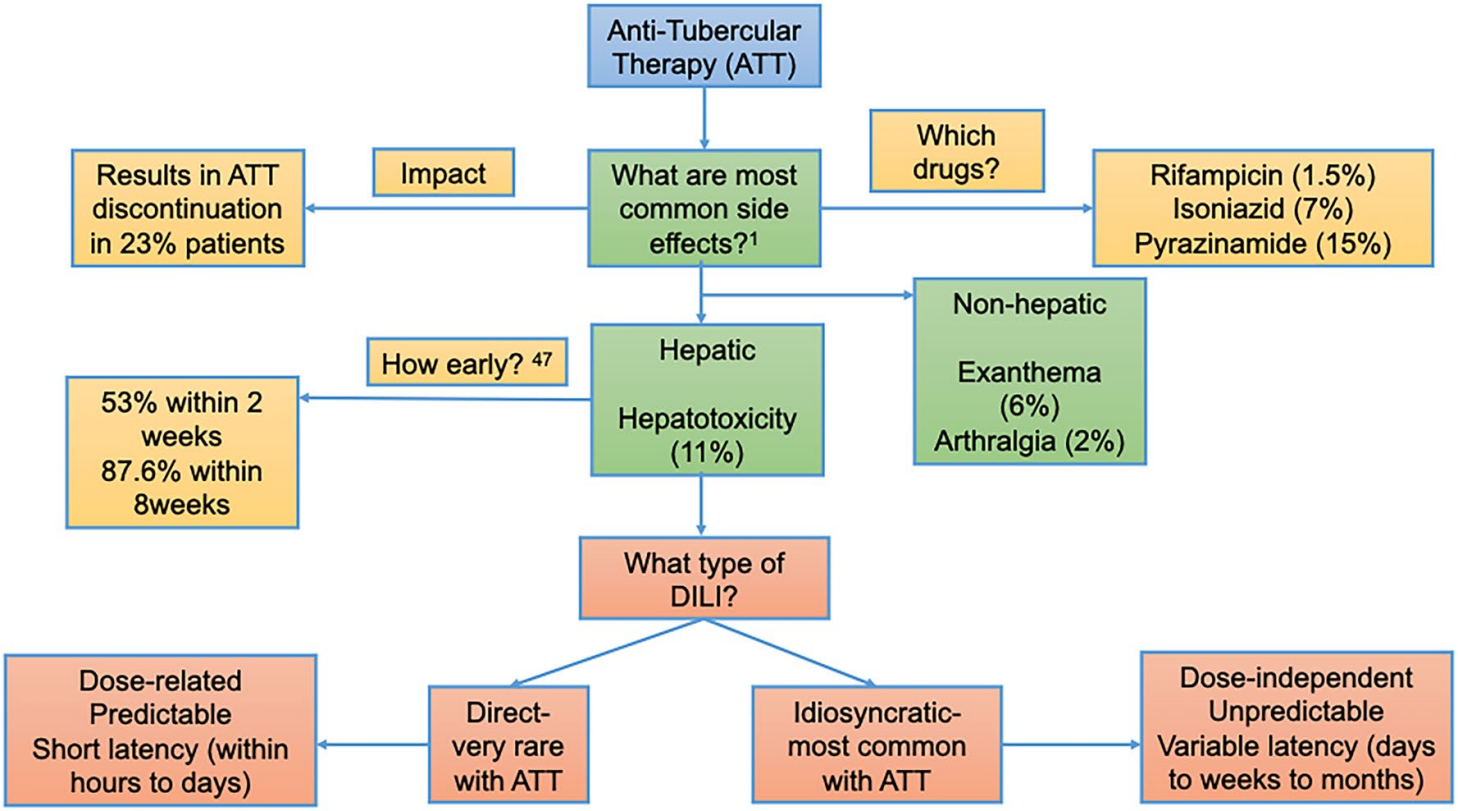
Importance of ATT‐DILI. Superscripts signify references as in text. DILI, drug‐induced liver injury.

Drug‐induced liver injury (DILI) is a major clinical problem in tuberculosis (TB) management. In retrospective analysis of 519 TB patients, hepatotoxicity was the commonest severe adverse effect of anti‐tubercular therapy (ATT), necessitating drug withdrawal in 11% of patients followed by exanthema (6%) and arthralgia (2%) [1] (Figure 1).

The spectrum of DILI ranges from acute hepatitis to acute liver failure (ALF) or acute on chronic liver failure (ACLF), ultimately leading to poor adherence to therapy, significant morbidity and mortality. The diagnosis of DILI is challenging due to the lack of specific diagnostic tests; rather, it is a diagnosis of exclusion by excluding infective and other potential noninfective causes with certainty.

Traditionally, DILI is classified as intrinsic (or direct) and idiosyncratic. Intrinsic DILI is due to drugs that are inherently toxic to the liver. This type of DILI is typically dose‐related, predictable, and onset is within hours to days (short time span). Injurious free radicals cause hepatocyte necrosis in zones farthest from the hepatic arterioles, where metabolism is greatest and antioxidant detoxifying capacity is the least [2].

Idiosyncratic DILI, on the other hand, is due to drugs that are not inherently toxic to the liver. This type of DILI is dose‐independent, unpredictable and has variable latency to onset of days to weeks to months. Hepatocyte necrosis is often distributed throughout hepatic lobules rather than being zonal, as is often seen with predictable DILI [3].

ATT‐related DILI (ATT‐DILI) is of idiosyncratic type and is the most common cause of idiosyncratic DILI worldwide [4]. Being a TB endemic zone, in many Asian countries, ATT is the most common cause of DILI and drug‐induced ALF [5, 6]. ATT is the second most common drug causing ACLF [7].

The incidence of ATT‐DILI is variable due to different definitions used for DILI across different time frames. The spectrum of ATT‐DILI ranges from hepatitis in 2.3% to 28% patients [8], to acute liver failure (ALF) in 25% with overall mortality and ALF mortality rate of 22.7% and 69.6%, respectively [6]. ALF due to ATT has a more aggressive course and constituted 5.7% of all ALF at a tertiary center with more than 60% mortality rate [9, 10].

It is, therefore, important to understand which ATT drugs cause hepatotoxicity and their metabolism leading to DILI in association with both modifiable and non‐modifiable predisposing factors, so that appropriate steps can be taken for prevention, early detection, and management of ATT‐DILI. Among first‐line ATT drugs, pyrazinamide is the most hepatotoxic, followed by isoniazid and rifampicin.

## 2 Anti‐Tubercular Drugs

### 2.1 Isoniazid

Isoniazid (INH) is cleared predominantly by the liver. Acetylation, oxidation, and hydrolysis are the three types of chemical reactions in the isoniazid metabolism pathway that are of clinical interest. Acetylation and oxidation are enzyme‐mediated, and hydrolysis is a non‐enzyme‐mediated reaction. Two key enzymes in the metabolic pathway are microsomal enzyme cytochrome P4502E1 (CYP2E1) and *N*‐acetyltransferase 2 (NAT2), and they determine the risk of hepatotoxicity. NAT2 is responsible for metabolism through acetylation of isoniazid finally to diacetyl hydrazine, which is non‐hepatotoxic. Cytochrome P4502E1 (by oxidation) and hydrolysis lead to the formation of hepatotoxic metabolites like acetyl‐diazine and other reactive acetyl onium ions and acetyl radicals, which covalently bind to cellular macromolecules and cause hepatocyte necrosis.

**FIGURE 2:**
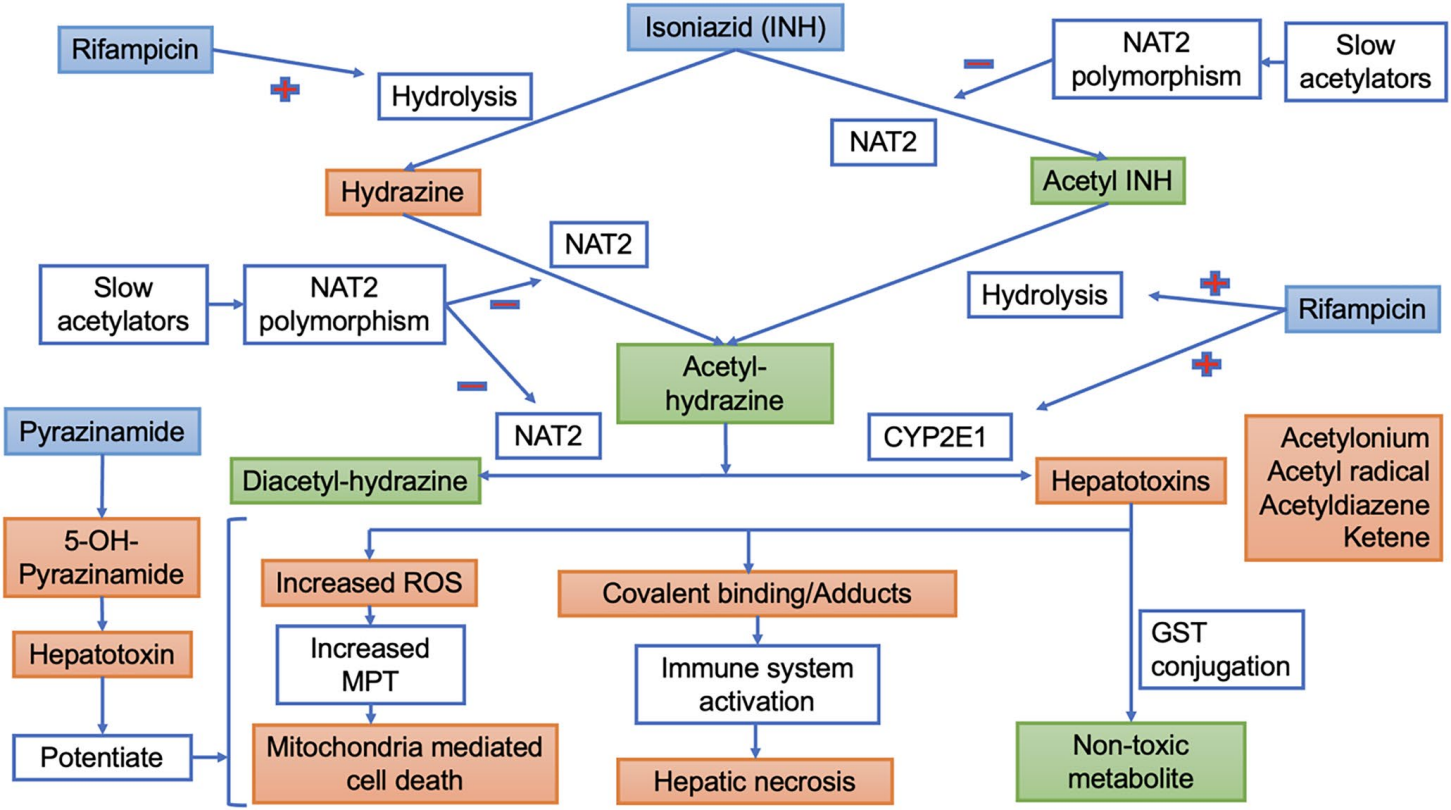
Pathway of hepatotoxicity by anti‐tubercular drugs. CYP2E1, cytochrome P4502E1; GST, glutathione S‐transferases; MPT, mitochondrial permeability transition; NAT2, *N*‐acetyltransferase 2; ROS, reactive oxygen species. Light orange‐colored boxes signify hepatotoxic metabolites. Light green‐colored boxes signify nontoxic metabolites. Light blue‐colored boxes signify hepatotoxic anti‐tubercular drugs. “+” signifies potentiation. “–” signifies inhibition.

Slow acetylation, as seen in certain NAT2 genotype individuals, is associated with accumulation of hepatotoxic intermediates and has four‐fold increased risk of isoniazid induced DILI as compared to controls with an odds ratio of 4.6. Individuals with CYP2E1 c1/c1 genotype were 2.5 times more likely to develop ATT‐DILI when compared with the other genotypes. Slow‐acetylator status (NAT2 gene polymorphism) and the CYP2E1 C/D or C/C genotype together showed a higher frequency in DILI [11]. Direct hydrolysis (minor pathway) of INH without acetylation leads to formation of hydrazine which is hepatotoxic. In slow acetylators and with concomitant rifampicin use, this minor hydrolysis pathway is increased 10‐fold, leading to DILI [12] (Figure 2).

Glutathione is an intracellular free radical scavenger and plays a protective role by sulfhydryl conjugation of the toxic reactive metabolites thereby facilitating their excretion from the body and decreasing hepatotoxicity. Deficiency in GST activity, because of homozygous null mutations at GSTM1 and GSTT1 loci, modulates susceptibility to drug‐ and xenobiotic‐induced hepatotoxicity. ATT‐DILI was 2.6 times more likely in those with GSTT1 null mutations [13].

### 2.2 Rifampicin

Rifampicin is metabolized in the liver by deacetylation and hydrolysis to nontoxic metabolites which are almost equally excreted in bile and urine. Rifampicin is associated with a hepatocellular pattern of idiosyncratic DILI [14, 15] mostly by potentiating the hepatotoxicity of other ATT drugs [16, 17] which is by virtue of its potent enzyme‐inducing property, particularly the cytochrome P450 (CYP3A4) system via the hepatocyte xenosensor, that is, pregnane X receptor (PXR) in the cell nucleus [18]. This leads to increased metabolism of isoniazid to toxic intermediate metabolites. Rifampicin also induces isoniazid hydrolysis minor pathway metabolism by 10‐fold, particularly in slow acetylators, leading to DILI [12].

Rifampicin excretion in bile occurs by transporter ABCB1. Dose‐dependent interference by saturation of the transporter protein is possible, leading to mild unconjugated hyperbilirubinemia without hepatotoxicity. However, any genetic variation in the gene coding this protein can result in increased accumulation of active metabolite backstream with enzyme‐inducing property and thus resultant TB‐DILI, as shown in a study where patients homozygous for ABCB1 3435TT genotype had a three‐fold increased risk of DILI with ATT and anti‐retroviral treatment [19, 20]. Rifampicin can also lead to mild isolated conjugated hyperbilirubinemia by inhibiting bile salt exporter pump (BSEP) and thus causing decreased conjugated bilirubin excretion [21].

### 2.3 Pyrazinamide

Pyrazinamide is a nicotinic acid derivative which is de‐amidated to pyrazinoic acid, which is further oxidized to 5‐hydroxy pyrazinoic acid (5‐OH‐PA) by xanthine oxidase and is finally excreted in urine. Xanthine oxidase also directly acts on pyrazinamide to form 5‐hydroxy pyrazinamide which is considered the main toxic metabolite responsible for hepatotoxicity; this is further de‐amidated to 5‐OH‐PA. Xanthine oxidase inhibitors such as allopurinol increase half‐life of pyrazinamide, which is also seen in chronic liver disease. These patients are more prone to dose‐dependent adverse effects with increased risk of idiosyncratic reactions. Half‐life of pyrazinamide is longer than isoniazid and rifampicin. Pyrazinamide can cause both idiosyncratic and dose‐dependent adverse reactions; the latter is less known to occur with the current regimen dosage. Pyrazinamide can also lead to granulomatous hepatitis and hypersensitivity reactions with eosinophilia [22].

### 2.4 Ethambutol

Ethambutol interferes with the biosynthesis of arabinogalactan in the cell wall and has synergistic effects with INH. This synergistic effect is through binding of ethambutol to a transcription factor encoded by the *Rv0273c* gene which leads to transcriptional repression of the inhA gene that encodes proteins necessary for cell wall integrity. This leads to enhanced INH sensitivity of the inhA gene and increased mycobactericidal effect of INH [23].

Being a bacteriostatic drug, it is always used in combination with other ATT drugs even for latent TB prophylaxis. Due to this, the amount of raised transaminases attributable to ethambutol alone is difficult to elicit. However, the addition of ethambutol to isoniazid, rifampin, or pyrazinamide does not appear to increase transaminases and hepatotoxicity. There has been one case report of ethambutol‐induced cholestatic pattern of liver injury which reoccurred after rechallenge, but the data provided were insufficient and circumstances unclear [24]. In conclusion, due to lack of high‐quality data, ethambutol is not considered a hepatotoxic drug.

### 2.5 Fluoroquinolones

Among commonly used fluoroquinolones, levofloxacin is excreted unchanged in urine and ciprofloxacin is metabolized in the liver. When hepatotoxic first‐line anti‐tubercular drugs are stopped, fluoroquinolones are added, which are per se second line ATT drugs in the context of MDR TB, and it has been observed that such addition of quinolones did not cause increased hepatitis [25]. Furthermore, use of ofloxacin in CLD is well tolerated and is effective. Adverse drug reactions are related to hypersensitivity to the drug which may result in hepatitis, eosinophilia, and febrile illness [26].

### 2.6 Linezolid

Linezolid is a potent second line ATT. With increasing experience, it is now being used in modified ATT regimens and is considered non‐hepatotoxic. Being a lipophilic drug, it is widely distributed and acts by inhibiting bacterial protein synthesis by preventing the fusion of ribosomal subunits. It is metabolized in the liver by oxidation of the morpholine ring to inactive compounds, without involvement of the cytochrome P450 system. Urinary excretion is a major elimination route. Linezolid has been associated with mild and transient elevations in serum aminotransferase and alkaline phosphatase levels in 1%–10% of patients, the mechanism of which is not known and may relate more to the underlying condition rather than injury from the drug per se [27]. This is important in patients with chronic liver disease and in lactic acidosis. The most common reasons for linezolid discontinuation in patients with tuberculosis are non‐hepatic adverse effects like myelosuppression, neurotoxicity, hyperlactatemia, and diarrhea, all of which are probably due to inhibition of human mitochondrial ribosomal function. Linezolid should not be given to patients on serotonergic agents. It is common practice to prescribe linezolid with vitamin B6 [28].

### 2.7 DILI With Other Anti‐TB Agents

Besides being an antibiotic with no hepatotoxic potential, cycloserine is also a GABA transaminase inhibitor and therefore has a risk of precipitation of alcohol withdrawal seizures in chronic alcoholics [29]. Ethionamide and prothionamide have a 2% risk and PAS has a 0.3% risk of hepatotoxicity [30] which are minimal. Bedaquiline causes mild to moderate asymptomatic LFT derangement in 8%–12% of patients which is mostly self‐limiting [31].

## 3 Pathophysiology

### 3.1 Role of Metabolite Accumulation

Most ATT drugs are lipid soluble. Hepatic metabolism of a liposoluble drug requires a series of biotransformation to a more water‐soluble compound to facilitate elimination from the body. This process of metabolism is divided into three phases. Phase 1 involves functionalization reactions like oxidation or demethylation which are usually performed by CYP450 enzymes. This results in partially water‐soluble and toxic intermediates. Phase 2 involves conjugation reactions like glucuronidation or sulfation which are usually performed via the glutathione detoxification pathway. This results in a more water‐soluble and nontoxic metabolite. Phase 3 refers to transporter‐mediated elimination of metabolites from the body.

The expression of enzymes and transporters involved in all the above phases of metabolism is regulated by transcription factors like pregnane X receptor and is influenced by the genetic and environmental factors which determine the rate of formation and accumulation of reactive metabolites. Phase 1 reaction leads to increased reactive metabolites. Dysfunctional Phase 2 and Phase 3 reactions lead to failure of detoxification and excretion of these reactive metabolites, respectively. This ultimately leads to cell death through lipid peroxidation caused by excessive reactive oxygen species [32].

### 3.2 Role of Cellular Environment

Hepatocyte can modulate its tolerance and adaptability to oxidative stress. Heterodimers of transcription factor nuclear factor erythroid 2‐related factor‐2 (Nrf2) and small Maf basic leucine zipper (bZIP) proteins upregulate expression of antioxidant pathway and are cyto‐protective, whereas this antioxidant pathway is downregulated by heterodimer complex of bric‐a‐brac (BTB) domain, cap'n'collar (CNC) type of basic region homology 1 (Bach1), and small Maf proteins. Increased downregulation and decreased upregulation of antioxidant pathway may be responsible for perpetuation of ATT‐DILI pathway [33].

### 3.3 Role of Mitochondrial Metabolism

Mitochondria play an important role in DILI. The inner mitochondrial membrane is the site of oxidative phosphorylation by respiratory chain complex which is inhibited by the reactive drug metabolites leading to ATP depletion and ultimately leading to increased generation of reactive oxygen species (ROS), increased oxidative stress and mitochondria‐mediated cell death. The mitochondrial matrix is the site of manganese superoxide dismutase (MnSOD) which scavenges superoxide anion. These ROS are generated constantly as a byproduct of electron transport in respiratory chain complex and their accumulation due to dysfunctional MnSOD leads to alteration in the permeability of inner mitochondrial membrane. This mitochondrial permeability transition may lead to necrosis due to decreased ATP generation, or may lead to apoptosis through release of pro‐apoptotic factors. A relationship between susceptibility to ATT‐DILI and genetic polymorphisms of this enzyme was shown by a study in which patients with ATT‐induced DILI having mutant C allele (T/C or C/C genotype) of MnSOD had a 2.5‐fold higher risk of hepatotoxicity when compared with those with MnSOD TT genotype [34].

### 3.4 Role of Adduct Mediated Immune Response

Under physiological conditions, it is normal for a drug to undergo metabolism to produce metabolites which may be capable of binding to cellular proteins forming adducts/neo‐antigens. These adducts are cleared subsequently without mounting an immune response in the majority of individuals but not in all. Individuals with susceptible specific class 2 HLA alleles can activate an adaptive immune response against adducts. There can be three types of outcomes following immune activation. First, the majority will have no hepatic injury due to strong tolerance. Second, a minority will have mild hepatic injury which is followed by adaptation with drug continuation. Third, very few susceptible patients will develop overt DILI due to inadvertent and aberrant T‐cell activation mediated end‐organ damage [35].

The risk of hepatotoxicity from anti‐tuberculosis drugs was more in individuals having of HLA‐DQB1*0201 allele (OR 1.9) and in individuals lacking HLA‐DQA1*0102 allele (OR 4.0). These MHC class 2 alleles were independent risk factors for ATT‐induced DILI [36].

### 3.5 Role of Cytokines/Chemokines

A co‐stimulatory molecule is required for effective interaction of antigen presenting cells and T cell receptors for mounting an adaptive immune response. This co‐stimulatory molecule can be cytokines released by damage associated molecular patterns (DAMPs) due to oxidative stress or can be released by chronically infected hepatic cells like in chronic viral hepatitis. Patients with chronic viral infections in the liver are at a risk for ATT‐DILI. This although manifests as sub‐clinical response but indicates vulnerability of an individual for overt DILI [37].

### 3.6 Role of Histone/Epigenetics

**FIGURE 3:**
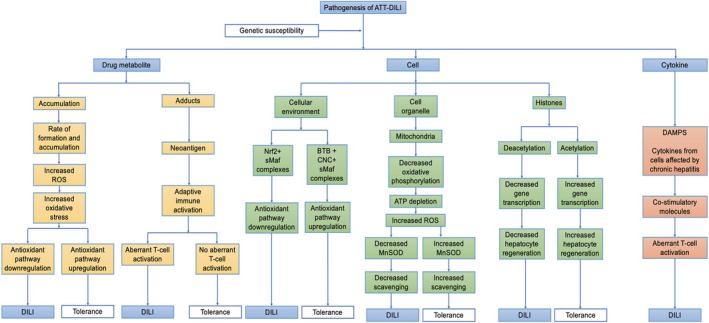
Pathogenesis of ATT‐DILI. ATP, adenosine triphosphate; BTB, bric‐a‐brac gene domain; CNC, cap'n'collar gene domain; DAMPs, damage associated molecular patterns; MnSOD, manganese superoxide dismutase; Nrf, nuclear factor erythroid 2‐related factor‐2; ROS, reactive oxygen species; sMaf, small Maf proteins.

Histones play an important role in gene regulation. Histone acetylation increases gene transcription through the promotor region and deacetylation decreases transcription. Acetylation of histones is mediated by the histone acetyltransferase enzyme. It has been postulated that the acetylation of drugs having hydralazine derivatives like isoniazid is also mediated by the histone acetyltransferase enzyme and prolonged use of such drugs leads to exhaustion of this enzyme leading to histone deacetylation and decreased transcription of factors involved in hepatic regeneration ultimately supporting DILI [38] (Figure 3).

## 4 Risk Factors

### 4.1 Non‐Modifiable Risk Factors

- Age: Reduced hepatic blood flow and altered metabolism with advancing age increase susceptibility to DILI [39]. Multiple studies show higher ATT‐DILI risk above 50–60 years, with severity and mortality also rising in older groups.
- Gender: Women have up to four‐fold greater susceptibility which is attributed to higher CYP3A4 activity. Higher risk has been reported in peripartum period [40, 41].
- Genetic predisposition: Asian men have greater susceptibility compared to other ethnic groups [42]. Variations in CYP3A4 and PXR genes, absence of *HLA‐DQA1*0102*, and presence of *HLA‐DQB1*0201* alleles are linked with increased risk.

### 4.2 Modifiable Risk Factors

- Nutrition: Malnutrition and fasting affect cytochrome P450 enzyme system which is responsible for metabolism of anti‐TB drugs [43]. Weight loss of more than 2 kg within 4 weeks of initiating ATT is a significant independent risk factor for DILI [44].
- Alcohol: Chronic alcohol use independently increases liver injury risk.
- Viral infections: Coinfection with HBV, HCV, or HIV increases both the frequency and severity of hepatotoxicity. ATT‐DILI is ~3–5 times more common in HBV/HCV‐infected patients and up to 14 times higher in HIV‐HCV coinfection [45].

## 5 Clinical Features

ATT‐related DILI has a broad spectrum, from asymptomatic raised transaminases to symptomatic hepatitis and acute liver failure (ALF). Differentiation from viral hepatitis is difficult; therefore, exclusion of other causes is essential. In most cases, hepatotoxicity improves after withholding therapy.

**FIGURE 4:**
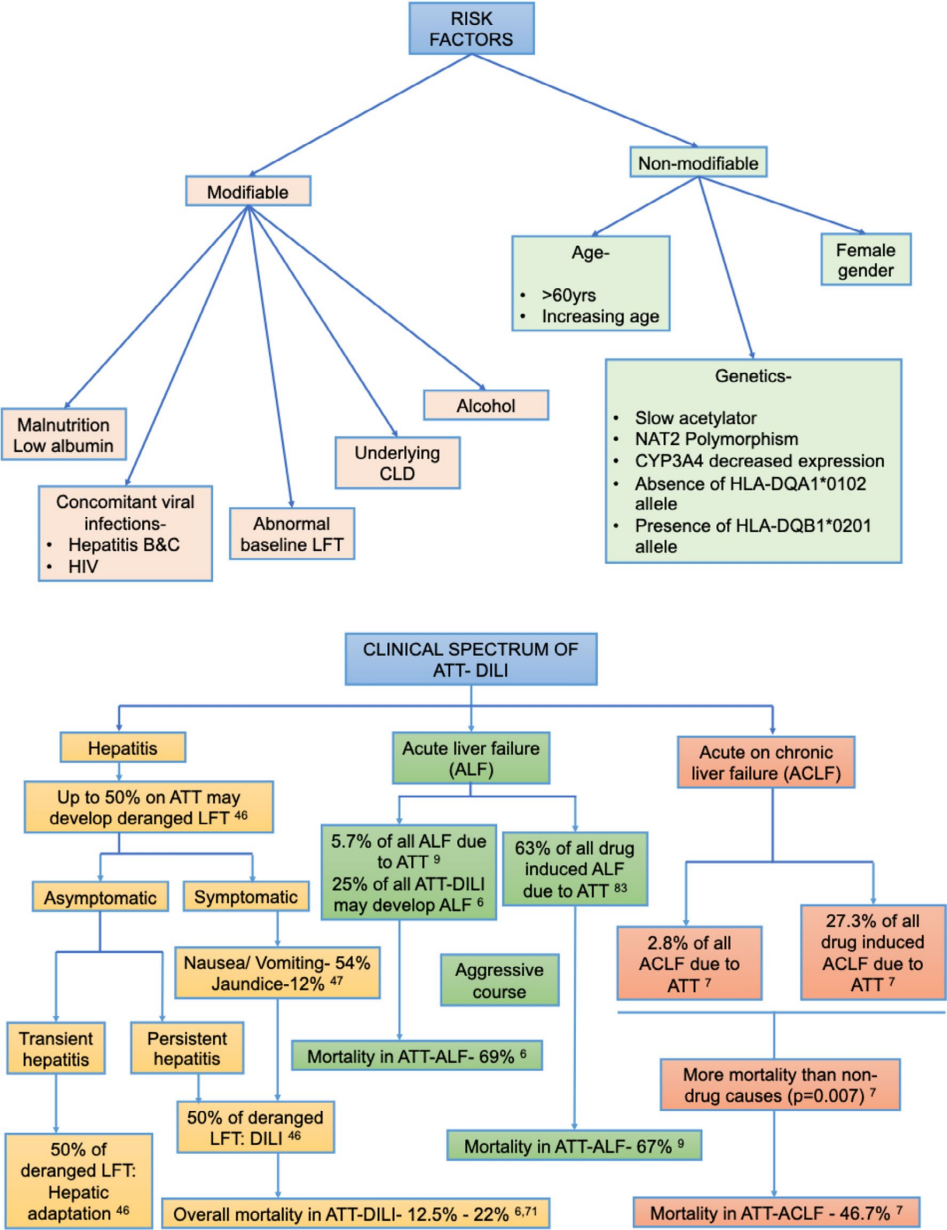
Risk factors and clinical spectrum of ATT‐DILI. Superscripts signify references as in text. CYP3A4, cytochrome P4503A4; LFT, liver function test; NAT2, *N*‐acetyltransferase 2.

Raised transaminases occur in 50% of patients on ATT [46]. About 50% of them develop mild, self‐limited raised transaminases which is termed hepatic adaptation [46]. In a large UK study, DILI occurred in 6.9% of TB patients; half within 2 weeks and nearly 90% within 8 weeks of ATT initiation. The most frequent symptoms were nausea and vomiting (50%), abdominal pain, and skin manifestations, while jaundice occurred in 12% [47]. Other series report nausea, vomiting, anorexia, and abdominal pain in up to 90% of cases [48] (Figure 4).

A recent systematic review and meta‐analysis found ATT‐DILI in 12.6% of patients, and ATT‐ALF in 7% with 70% mortality [49]. Mean latency from ATT initiation to ALF was 30 days and independent predictors of mortality were bilirubin ≥ 10.8 mg/dL, PT ≥ 26 s, and Grade III–IV encephalopathy [9].

ALF due to ATT differs from viral hepatitis related ALF. It usually has subacute onset, disproportionately elevated bilirubin and INR, mixed‐pattern liver injury, and poorer prognosis due to high mortality and TB treatment challenges. By contrast, viral hepatitis ALF presents with abrupt onset, higher transaminase elevations, and generally better recovery.

## 6 Diagnosis of ATT‐Induced Hepatitis

Alkaline phosphatase (ALP) thresholds are less reliable in TB, since granulomatous hepatitis itself may elevate levels. Mild aspartate aminotransferase (AST) or alanine aminotransferase (ALT) elevation during ATT can also reflect hepatic adaptation. Isolated bilirubin elevation is rare in ATT‐DILI and usually reflects rifampicin induced transient, benign unconjugated hyperbilirubinemia without hepatocellular injury. Significant bilirubin rise almost always occurs with ALT/AST elevation. Thus, distinguishing benign changes from DILI is essential to avoid premature drug withdrawal while ensuring timely recognition of clinically significant hepatotoxicity, which carries high mortality.

According to the American Thoracic Society (ATS) guidelines and widely accepted expert recommendations, ATT‐induced hepatotoxicity is defined as: (1) AST or ALT ≥ 3 times the upper limit of normal (ULN) with clinical symptoms suggestive of hepatotoxicity (nausea, vomiting, abdominal pain, jaundice, or unexplained fatigue) or (2) AST or ALT ≥ 5 times the ULN without symptoms.

In addition, other causes should be excluded and there should be improvement in AST/ALT after cessation of hepatotoxic ATT.

## 7 Management of ATT‐Dili

The guidelines for ATT‐induced hepatotoxicity come from the American Thoracic Society (ATS) [22] the British Thoracic Society (BTS) [50], the World Health Organization (WHO) [51], the Asian Pacific Association for the Study of the Liver (APASL) [52], and the Revised national tuberculosis control program (RNTCP) in India.

### 7.1 Pretreatment Evaluation for TB‐DILI

All patients should be screened for conditions that might have predisposition for ATT‐induced hepatotoxicity. This assessment mainly focuses on detecting any baseline hepatitis or presence of chronic liver disease, if any, prior to initiation of ATT. History of high‐risk sexual behavior, intravenous drug abuse, chronic ethanol consumption, prior history of chronic viral hepatitis, jaundice and history of use of concomitant potential hepatotoxic drugs/complementary and alternate medicines (CAM) should always be elicited. Assessment for malnutrition is important. If history is suggestive of the possibility of underlying liver disease, appropriate assessment should be done accordingly. It is recommended to get baseline serological evaluation including LFT and chronic viral infection markers (HIV, hepatitis B and C) [50, 53]. Taking into consideration the known risk factors for ATT‐DILI, it is very important to assess risk‐benefit ratio before starting empirical treatment as studies have shown more adverse drug reactions in them [9].

If ATT is to be given for latent TB, then it is preferable to start a monotherapy with isoniazid (first preference) or rifampicin (second preference due to drug interactions by virtue of its CYP enzyme‐inducing property). The combination therapy has been shown to be associated with a higher risk of hepatotoxicity. The risk of clinical hepatitis with isoniazid alone, rifampicin alone, and with combination therapy of both is 0.6%, 0%, and 2.73%, respectively [22, 54, 55].

If ATT is to be given in an individual with baseline liver disease, then ATT should consist of fewer hepatotoxic drugs depending upon the severity of liver disease [51, 55] (Table 1). Patients with Child Turcotte Pugh (CTP) scores of 7 or less generally tolerate two hepatotoxic drugs, whereas, those with scores between 8 and 10 are able to tolerate only one [56]. Based on experts' opinion, oral linezolid can be added as a substitute to injectable aminoglycosides in Child's class A and B liver cirrhosis. There is no adequate dose adjustment data of linezolid for Child's class C liver cirrhosis. Some authors recommend the use of a low dose of linezolid (600 mg/day) for maintaining adequate trough level in patients with Child's class C liver cirrhosis [57]. Therapeutic drug monitoring of linezolid should be done whenever feasible. Myelosuppression (particularly thrombocytopenia) should be monitored particularly in the background of chronic liver disease.

**TABLE 1:** Modified ATT regimens in ATT‐DILI and in CLD.

Modified ATT regimens in ATT‐DILI and in CLD [55, 56]				
Regimens	Drugs and duration	CTP score	Liver disease	Comments in CLD
Two hepatotoxic drugs regimen				
Regimen without isoniazid	6–9 months of rifampicin, pyrazinamide, and ethambutol	< 7	Stable	Less preferable; rarely used^c^
Regimen without pyrazinamide	9 months of isoniazid and rifampicin, plus ethambutol	< 8	Stable	Avoid pyrazinamide
	2 months of isoniazid, rifampicin, streptomycin/linezolid^a^ and ethambutol, followed by 6 months of isoniazid and rifampicin			
One hepatotoxic drug regimen	2 months of isoniazid (or rifampicin), ethambutol and streptomycin/linezolid^a^, followed by 10 months of isoniazid and ethambutol	8–10	Advanced	Rifampicin is preferred over isoniazid. Pyrazinamide should not be used
No hepatotoxic drug regimen	18–24 months treatment with a combination of ethambutol, fluoroquinolone, cycloserine, and capreomycin or aminoglycoside/linezolid^b^	> 10	Very advanced	If improvement in CTP is persistent, above regimens may be considered on case‐to‐case basis

Abbreviations: CLD, chronic liver disease; CTP, Child Turcotte Pugh score.

^a^ Indicate that oral linezolid can be used instead of streptomycin but has still not been positioned in guidelines.

^b^ Indicates that dose of oral linezolid needs to be modified (600 mg/day) and therapeutic drug monitoring should be considered.

^c^ May be used if only isoniazid has proven idiosyncratic DILI and pyrazinamide has proven good tolerance in a particular patient.

### 7.2 Monitoring for Early Detection of ATT‐DILI

Early detection by frequent monitoring is associated with better prognosis and lower mortality. In situations where baseline LFT is normal, almost all the guidelines do not recommend LFT monitoring unless the patient is symptomatic. However, in routine clinical practice, it is reasonable to monitor LFT every 2–4 weeks during the first 1–2 months, since slow acetylator status cannot be predicted. If baseline LFT is deranged, the frequency of LFT monitoring on ATT should be weekly if baseline ALT is > 2 times ULN and 2–4 weekly if baseline ALT is < 2 times ULN. If underlying liver disease is present, then LFT should be monitored weekly at least for 2 months, followed by clinical monitoring on a case‐to‐case basis.

### 7.3 Intervention for ATT‐DILI

Prompt withdrawal of all the potential hepatotoxic drugs is the key step in the management of DILI after diagnosis. In the absence of symptoms attributable to hepatotoxicity, hepatotoxic ATT (Isoniazid, rifampicin, and pyrazinamide) should be stopped only if ALT is > 5 times ULN. Mild raised transaminases to less than five times ULN in asymptomatic individuals may represent physiological hepatic adaptation to ATT and is expected to resolve with continuation of therapy. In the presence of symptoms attributable to hepatotoxicity, hepatotoxic ATT should be stopped if ALT is > 3 times ULN. It is important to note that in the absence of a specific biomarker that can differentiate between hepatic adaptation and potential DILI, the cut‐offs for stopping hepatotoxic ATT are based more on practical experiences rather than evidence based.

Increase in bilirubin with ATT should be taken as a guide to evaluate whether it is related to worsening of hepatic functions due to hepatotoxicity of drugs as suggested by concomitant raised transaminases and deranged INR. Else, isolated hyperbilirubinemia does not require modification of therapy but careful monitoring should be done [47, 50].

After ATT‐DILI diagnosis, all first‐line ATT except ethambutol should be stopped and other non‐hepatotoxic ATT like levofloxacin with either aminoglycoside (injectable) or linezolid/cycloserine (oral) should be added. Since there are no recent guidelines for choosing non‐hepatotoxic ATT in this scenario, the choice of ATT, besides ethambutol, depends upon the physician's preference and experience. Therapy can also be individualized depending upon the patient's preference of injectable or oral drug. A total of at least three non‐hepatotoxic drug‐based ATT regimens should be given during this time till the likely culprit drug is identified.

### 7.4 Restarting ATT

Hepatotoxic drugs should be withheld ideally till ALT normalizes or at least till ALT decreases to < 2 times ULN [54]. Since the first‐line ATT drugs are well known to be highly efficacious, the benefits of reintroduction of these drugs outweigh the closely monitored risk of hepatotoxicity with rechallenge [58]. Sharma et al. found that re‐exposure to the same ATT drug regimen leads to recurrence of DILI in only 11%–24% of patients which is not affected by the degree of initial injury [59].

Since more than one first‐line ATT is potentially hepatotoxic, reintroduction can be done in two ways: either sequential or concomitant, with either starting with full dose or incremental dose of individual drugs. A new drug should be reintroduced only if LFT is normal or < 2 times ULN. The first hepatotoxic drug to be reintroduced varies with different guidelines. The ideal regimen should be sequential with incremental dosage starting with least hepatotoxic drug first, but evidence for this is lacking. Since hepatotoxicity rate does not differ significantly between different regimens, reintroduction is often individualized guided by perceived clinical risk [59]. ATS and BTS favor sequential regimen. WHO recommends starting all drugs simultaneously and if a second episode of DILI occurs, then to follow sequential regimen. ATS recommends reintroducing rifampicin full dose first followed by isoniazid full dose after 3–7 days followed by pyrazinamide only if the initial injury was mild. BTS recommends isoniazid incremental dose first followed by rifampicin incremental dose followed by pyrazinamide. APASL recommends a combination of two regimens, that is, reintroducing rifampicin in incremental dose first followed by Isoniazid in incremental dose followed by pyrazinamide only if the initial injury was mild. RNTCP advocates use of any these regimens with rifampicin first; however, sequential full‐dose regimen is mostly practiced with repeat LFT every week to find the culprit drug. If ATT‐induced hepatotoxicity is severe (ALF or coagulopathy), pyrazinamide reintroduction should be avoided if rifampicin and isoniazid are well tolerated.

A landmark randomized study of 175 patients with ATT‐DILI found no difference in the recurrence rate of hepatotoxicity with three different reintroductory regimens of ATT (arm 1 with concomitant regimen including pyrazinamide, arm 2 with sequential regimen according to ATS, and arm 3 with sequential regimen according to BTS). Another randomized controlled trial of 45 patients with ATT‐DILI found that sequential reintroduction regimen without pyrazinamide had better safety than the concomitant regimen including pyrazinamide. An individualized approach based on risk factor assessment is needed. Malnutrition, HIV positivity, chronic viral hepatitis, low albumin, and chronic alcoholics have a higher risk of hepatotoxicity and may benefit more from sequential regimen with or without pyrazinamide [59].

**FIGURE 5:**
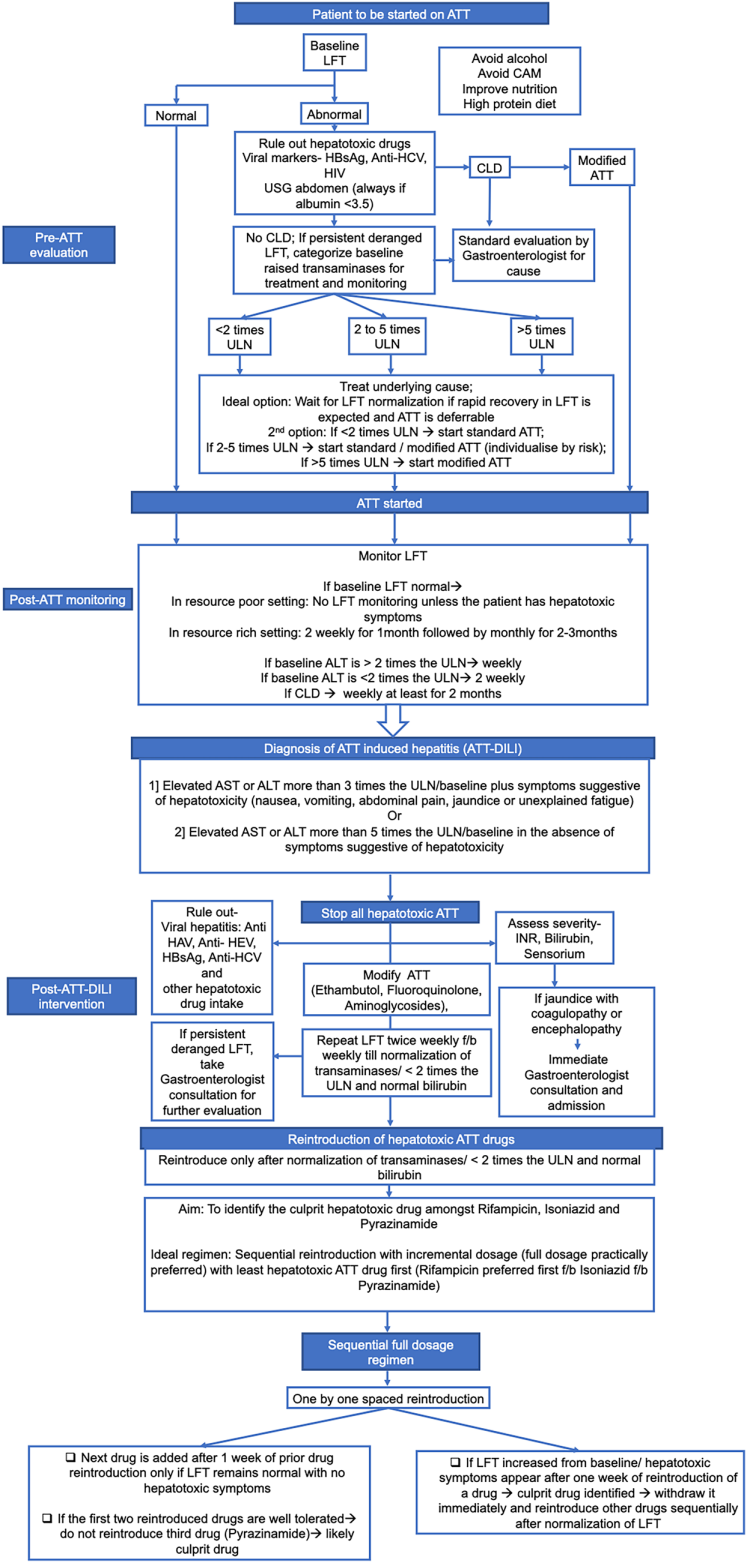
Pathway for management of ATT‐DILI. CAM, complementary and alternative medicines; CLD, chronic liver disease; f/b, followed by; HAV, hepatitis A; HBsAg, hepatitis B surface antigen; HCV, hepatitis C; HEV, hepatitis E; INR, international normalized ratio; LFT, liver function test; ULN, upper limit of normal; USG, ultrasound.

A systematic review and network meta‐analysis of four RCTs comprising over 500 patients found that when compared to concomitant regimen, the ATT‐induced hepatitis was significantly less in sequential and incremental regimen, and there was no difference in the rate of ATT‐induced hepatitis with rifampicin first or isoniazid first regimen [60] (Figure 5). Major studies on ATT‐DILI are summarized in Table 2.

**TABLE 2:** Summary of major studies on anti‐tuberculosis drug related hepatotoxicity.

Author/year	Country	Study design	Study population and setting	Patients on ATT	ATT‐DILI	Diagnostic criteria	Time to onset of DILI	Outcome measures	Key findings	Practical implications
Tahaoğlu et al. 2001 [61]	Turkey	RCT	ATT‐DILI patients requiring reintroduction of ATT; single‐center	45	45	LFT normalization after ATT withdrawal + ≥ 1: AST/ALT ≥ 5× ULN; or Bil. > 1.5 mg/dL; or any rise in AST/ALT with hepatitis symptoms; or exclude viral hepatitis	—	Recurrence rate of ATT‐DILI with incremental versus concomitant reintroduction regimen	Incremental regimen without pyrazinamide better than concomitant full‐dose regimen with pyrazinamide (DILI = 0% vs. 24%; *p* < 0.05)	Early evidence comparing reintroduction regimens
Sharma et al. 2010 [59]	India	RCT	ATT‐DILI patients needing rechallenge at tertiary center	175	175	LFT normalization after ATT withdrawal + ≥ 1: AST/ALT ≥ 5× ULN; or Bil. > 1.5 mg/dl; or any rise in AST/ALT with hepatitis symptoms; or exclude viral hepatitis	Median: 23 days (IQR: 4–44 days) Normalization time Median: 18 days (IQR: 14–28 days)	DILI with 3 reintroduction regimens: full‐dose simultaneous versus ATS versus BTS	No difference in DILI Reintroduction to DILI ~18 days (range: 5–35 days)	All major rechallenge protocols considered safe
Azuma et al. 2013 [62]	Japan	RCT	Newly diagnosed TB stratified by NAT2 acetylator genotype; Multicenter	172	14	ALT > 2× ULN; or ALP > 2× ULN; or mixed pattern + causality (RUCAM)	—	Isoniazid DILI and treatment failure in standard versus pharmacogenetic based treatment	Within 8 weeks‐DILI in 78% patients on standard treatment NAT2‐guided dosing reduced isoniazid DILI and failures	Proof of pharmacogenetic dosing utility
Zuberi et al. 2014 [63]	Pakistan	RCT	ATT‐DILI patients requiring reintroduction of ATT	325	325	Exclusion of other causes + ≥ 1: ALT > 5× ULN on one occasion; or ALT > 3× ULN on three consecutive occasions; or Bil. > 1.5 mg/dL	—	Recurrence rate of ATT‐DILI with incremental versus sequential full‐dose reintroduction regimen	No significant difference between two reintroduction strategy	Both the regimens are efficacious
Meena et al. 2016 [64]	India	RCT	ATT‐DILI patients requiring reintroduction of ATT	32	32	ALT ≥ 5× ULN, or ALT ≥ 3× ULN with bilirubin > 2× ULN, or ALP ≥ 2× ULN (with increased GGT)	—	Recurrence rate of ATT‐DILI with sequential isoniazid first versus sequential rifampicin first versus concomitant regimen (isoniazid and rifampicin)	No significant difference between three reintroduction strategy	All reintroduction strategy with isoniazid and rifampicin have similar efficacy
Tweed et al. 2018 [65]	Multi‐country	RCT dataset (post hoc)	Pulmonary TB patients enrolled in trial	1928	58	ALT ≥ 5× ULN or ALT ≥ 3× ULN with Bil. > 2× ULN + causality (RUCAM)	Median: 4 weeks (IQR: 2–8 weeks)	Risk factors, onset of DILI	Increased risk‐Isoniazid, older age, Asian ethnicity, HIV +ve. 3/4th DILI in 2 months	Frequent LFT monitoring in intensive phase
Moosa et al. 2021 [66]	South Africa	RCT	HIV‐prevalent hospitalized ATT‐DILI; multicenter	102	102	ATS criteria: AL ≥ 5× ULN (asymptomatic) or ALT ≥ 3× ULN (with symptoms) + exclusion of other causes	Median: 3 weeks (IQR: 10–40 days)	N‐acetylcysteine (NAC) versus placebo; ALT, hospital stay, mortality	NAC shortened length of stay; no difference in biochemical recovery and mortality	NAC may help as inpatient adjunctive therapy
Cai et al. 2012 [67]	China	Meta‐analysis	ATT tolerant (controls) and intolerant (DILI) patients	7131	2225	Heterogenous	—	Genetic risk	Slow NAT2, GSTM1 null, CYP2E1*1A genotype‐ increased risk of DILI	Predicting clinical response with genetic risk
Wang et al. 2012 [68]	China	Meta‐analysis	ATT tolerant (controls) and intolerant (DILI) patients	1920	474	Heterogenous	—	Genetic risk	NAT2 slow acetylators‐ increased risk of DILI	Predicting clinical response with genetic risk
Soni et al. 2020 [60]	India	Systematic review and network meta‐analysis	ATT‐DILI patients requiring reintroduction of ATT	577	577	Heterogenous	—	Recurrence rate of ATT‐DILI with different reintroduction regimen	Probability of recurrence of DILI: Incremental > Sequential > Concomitant regimen; but difference not significant. Rifampicin first versus Isoniazid first, similar DILI risk after reintroduction	All reintroduction strategy similar. Choice may be individualized with perceived risk
Wang et al. 2022 [69]	China	Systematic review and meta‐analysis	—	116 147	~12 776	Heterogenous	—	Incidence, trend, predictors	Global pooled ATT‐DILI incidence ~11%; upward trend over decades	Benchmark incidence with temporal trends
Akkahadsee et al. 2023 [70]	Thailand	Network meta‐analysis	Patients on ATT + hepatoprotective agents/placebo	3423	—	Heterogenous	—	Prevention of DILI (intervention versus placebo)	NAC, turmeric plus *Tinospora cordifolia* significantly decreases DILI incidence	Evidence certainty is limited
Kumar et al. 2025 [49]	India	Systematic review and meta‐analysis	TB patients	12 041	~1518	ALT/AST ≥ 5× ULN (asymptomatic) or ALT/AST ≥ 3× ULN (with symptoms) + exclusion of other causes	Median: 10–65 days	Incidence, risk factors	DILI: 12.6%; ALF ~7%; pooled mortality‐1.72%; ALF mortality ~70%; Increased risk: daily > intermittent regimen, HBV, HCV, alcohol, CLD, female, old age, low BMI, low albumin, slow acetylator	Intensify monitoring in India
Singh et al. 1996 [71]	India	Prospective cohort	ATT‐DILI patients at tertiary center	72	72	LFT normalization after ATT withdrawal + ≥ 1: AST/ALT ≥ 5× ULN; or Bil. > 1.5 mg/dL; or any rise in AST/ALT with hepatitis symptoms; or exclude viral hepatitis	Mean ± SD: 7 ± 5 weeks versus 5 ± 4 weeks (mortality vs. survival group)	Clinical and ATT reintroduction outcome	Jaundice‐61%, Prodomal‐39% Hepatic failure ~17% Mortality‐12.5% Reintroduction safe after recovery (41/44)	Early evidence supporting reintroduction of ATT
Agal et al. 2005 [72]	India	Prospective cohort	ATT tolerant and intolerant patients at tertiary center	224	45	Exclusion of viral, alcohol, drug causes + ≥ 1: symptoms of hepatitis; or AST/ALT > 5× ULN, or > 2× ULN with symptoms; or Bil. > 2× ULN	Mean ± SD: 12.6 ± 3.6 days	Monitoring and reintroduction strategy	Mortality ~17%. Sequential full‐dose reintroduction with weekly LFT successful strategy (38/39)	Guidance on reintroduction strategy
Kumar et al. 2010 [9]	India	Prospective cohort	ALF patients (*n* = 1223) at tertiary center	85	85	Jaundice + encephalopathy ≤ 4 weeks	ATT to encephalopathy Median (range): 4 weeks (1–50 weeks)	Incidence, mortality, predictors	ATT‐ALF‐5.7%; ATT‐ALF mortality ~67%; bilirubin (> 10.8), INR (PT > 26), encephalopathy independently predicted mortality	ATT‐ALF an important entity; early referral to transplant setting needed
Shang et al. 2011 [73]	China	Prospective cohort	Patient on ATT; multicenter	4304	106	AST/ALT ≥ 3× ULN; or Bil. > 2× ULN + exclusion of other causes	Median: 52 days (IQR: 30–63 days)	Clinical and treatment outcomes	Nausea (41%), vomiting (40%), anorexia (25%); asymptomatic DILI‐33%; mortality‐1.8%; 70% changed treatment; 50% transient cessation of ATT	Up to 1/3rd with DILI can be asymptomatic highlighting importance of monitoring
Singanayagam et al. 2012 [74]	UK	Prospective cohort	Patient on ATT at tertiary center	288	21	ATS criteria: ALT ≥ 5× ULN (asymptomatic) or ALT ≥ 3× ULN (with symptoms) + exclusion of other causes	57% DILI ≤ 2 weeks; 43% DILI > 2 weeks	Early versus late DILI in two monitoring approaches	LFT every two weekly promptly identifies early DILI better than ATS risk‐factor based monitoring	Guides monitoring strategy for patients on ATT
Gupta et al. 2013 [75]	India	Prospective cohort	Patient on ATT; multicenter	215	50	ALT ≥ 2× ULN, or a combined increase in AST and bilirubin levels with either ≥ 2× ULN	—	Gene polymorphism in ATT‐DILI versus No DILI	Slow‐acetylator NAT2 polymorphism‐ increased susceptibility to isoniazid DILI	Genetic risk profiling may lead to precision medicine
Rana et al. 2014 [76]	India	Prospective cohort	Patient on ATT at tertiary center	300	55	AST/ALT ≥ 5× ULN, or AST/ALT ≥ 2× ULN + increased bilirubin	—	Gene polymorphisms in ATT‐DILI versus No DILI	Slow‐acetylator genotypes (NAT2 5/7, 6/7) and GSTM1 allele of GST enzyme‐ increased risk of DILI	Genetic risk profiling may lead to precision medicine
Singla et al. 2014 [77]	India	Prospective cohort	Patient on ATT at tertiary center	408	17	AST/ALT ≥ 2× ULN		Gene polymorphism in ATT‐DILI versus No DILI	Increased risk: slow‐acetylator NAT2 genotypes, GSTM1 and GSTT1 double null genotype, heterozygote genotype “c1c2” of CYP2E1	Genetic risk profiling may lead to precision medicine
Satyaraddi et al. 2014 [78]	India	Prospective cohort	Patient on ATT at tertiary center	110	15	ALT/AST ≥ 5× ULN (asymptomatic) or ALT/AST ≥ 3× ULN (with symptoms) or ALT/AST ≥ 3× ULN with Bil. > 2× ULN + exclusion of other causes	Median (range): 22 days (10–35 days)	Serial plasma drug levels of isoniazid, rifampicin and pyrazinamide	Only rifampicin drug levels correlated with subsequent development of DILI	First mechanistic study of its kind. Drug levels of isoniazid and pyrazinamide cannot predict DILI
Gaude et al. 2015 [79]	India	Prospective cohort	Patient on ATT at tertiary center	3900	150	LFT normalization after ATT withdrawal + ≥ 1: AST/ALT ≥ 5× ULN; or Bil. > 1.5 mg/dL; or any rise in AST/ALT with hepatitis symptoms; or exclude viral hepatitis	Mean 20 days; lasted for 14 days	Incidence, risk factors	DILI‐3.8%; risk factors‐advanced age, hypoalbuminemia, regular alcohol intake and radiologically advanced nature of the disease	Regional variation in ATT‐DILI across different studies
Bright‐Thomas et al. 2016 [80]	UK.	Prospective cohort	Patient on ATT; multicenter	2070	63	ALT ≥ 5× ULN, or rising bilirubin	—	DILI incidence and trend over 30 years	DILI‐3%; incidence increased with age; presumed culprit: Pyrazinamide‐57%, Rifampicin‐32%, Isoniazid‐11%, Ethambutol‐0%. No increasing trend in incidence over time	Incidence and its trend have geographical variations
Latief et al. 2017 [81]	India	Prospective cohort	Patient on ATT at tertiary center	200	16	ATS criteria: ALT ≥ 5× ULN (asymptomatic) or ALT ≥ 3× ULN (with symptoms) + exclusion of other causes	63% DILI ≤ 2 weeks	Risk factors, monitoring outcomes	Increased risk‐Female, extra‐pulmonary TB. Early LFT monitoring at second, fourth, and eighth week leads to early DILI detection, irrespective of risk factors	LFT based early monitoring in all is better than risk‐factor based approach
Bessone et al. 2019 [82]	Latin America	Prospective registry cohort	All cause idiosyncratic DILI (*n* = 468)	—	20	ALT ≥ 5× ULN, or ALT ≥ 3× ULN with bilirubin > 2× ULN, or ALP ≥ 2× ULN (with increased GGT) + causality (RUCAM)	—	Etiology	ATT‐DILI (4.3%) less frequent	Regional variation in ATT‐DILI
Devarbhavi et al. 2021 [83]	India	Prospective registry cohort	All cause DILI patients (*n* = 1228); multicenter	—	~565	ALT ≥ 5× ULN, or ALP ≥ 2× ULN, or ALT ≥ 3× ULN with Bil. > 2× ULN + causality (RUCAM)	—	Etiology and overall clinical outcomes	46% ATT‐DILI; high hospitalization rate	Significant burden of ATT‐DILI in India
Jiang et al. 2021 [84]	China	Prospective cohort	Patients on ATT (derivation + validation cohorts)	4652	255	ALT ≥ 5× ULN, or ALP ≥ 2× ULN, or ALT ≥ 3× ULN with Bil. > 2× ULN	Median: ~42 days	Incidence, clinical outcomes	5.5% DILI, 0.8% mortality Increased risk‐older age, hepatitis B, high baseline ALT	Monitor high‐risk groups
Raj Mani et al. 2021 [85]	India	Prospective cohort	Patients on ATT	393	38	ALT ≥ 5× ULN (asymptomatic) or ALT ≥ 3× ULN (with symptoms) or Bil. > 1.5 mg/dL rise + exclusion of other causes	—	Incidence, risks, predictor score for ATT‐DILI	DILI‐ 9.7%, mortality ~5%, clinical risk score with HIV, CLD, daily treatment regimen, disseminated disease, undernutrition, female gender (sensitivity‐74%, specificity‐ 67%)	The scoring system needs validation
Moosa et al. 2023 [86]	South Africa	Prospective nested cohort	ATT‐DILI patients receiving NAC; multicenter	45	45	ATS criteria: ALT ≥ 5× ULN (asymptomatic) or ALT ≥ 3× ULN (with symptoms) + exclusion of other causes	Median: 18 days (IQR: 10–31 days)	Biomarker (miR‐122)	miR‐122 correlated with ALT; unaffected by NAC	Promising biomarker for ATT‐DILI
Lim et al. 2023 [87]	Korea	Prospective cohort	Patients on ATT; multicenter	684	52	ALT/AST ≥ 200 U/L or ≥ 120 U/L with symptoms; or Bil. ≥ 3 mg/dL or ≥ 1.5 mg/dL with symptoms	Median (range): 8 weeks (4–18 weeks)	Risk (presence or absence of metabolic disorder)	DILI‐7.6%. Metabolic comorbidities increase risk	Closer monitoring in metabolic syndrome
Petros et al. 2025 [88]	Ethiopia	Prospective cohort	Patients on ATT; multicenter	219	35	ALT ≥ 5× ULN, or ALT ≥ 3× ULN with Bil. > 2× ULN, or ALP > 2× ULN	75% DILI ≤ 4 weeks	Incidence, risk factors	ATT‐DILI incidence ~16% Female, old age, low BMI, HIV increased risk	Similar risk factors across nations
Kumar et al. 2025 [46]	India	Prospective cohort	Newly diagnosed abdominal TB patients; single‐center	140	20	ALT ≥ 5× ULN, or ALT ≥ 3× ULN with bilirubin > 2× ULN, or ALP ≥ 2× ULN (with increased GGT) + RUCAM	Median: 12 days (IQR: 7–22.7) 90% DILI ≤ 8 weeks	Clinical outcome, reintroduction success	LFT abnormalities‐50.7%; Median time (ATT to deranged LFT): 12 days (IQR: 7–22.7). Half recovered spontaneously (hepatic adaptation); median (deranged LFT to hepatic adaptation): 21 days (IQR: 14–42) DILI‐27%; low albumin/Vit D predicted progression to DILI; ~66% rechallenge success	Pattern of LFT changes under ATT
Sharma et al. 2002 [36]	India	Retrospective cohort	ATT tolerant and intolerant patients at tertiary center	346	56	LFT normalization after ATT withdrawal + ≥ 1: AST/ALT ≥ 5× ULN; or Bil. > 1.5 mg/dL; or any rise in AST/ALT with hepatitis symptoms; or exclude viral hepatitis	Median (range): 4 weeks (1–72 days)	Clinical and genetic risk factors	Increased risk‐alcohol, older age, low serum albumin, underlying liver disease, HLA‐DQA10102 absence, HLA‐DQB10201 presence	Genetic risk profiling
Devarbhavi et al. 2013 [6]	India	Retrospective	ATT‐DILI patients in tertiary center	269	269	Exclusion of other causes + ≥ 1: Bil. ≥ 2 mg/dL, or AST/ALT > 3× ULN, or ALP > 2× ULN + causality (RUCAM). ALF = jaundice + encephalopathy ≤ 8 weeks	Median (range): 2 months (4 days‐16 months)	ALF %, mortality, predictors	ALF ~25%, Overall mortality ~23%, ATT‐ALF mortality‐70% 5‐factor prognostic score (bilirubin, INR, creatinine, albumin and encephalopathy; AUROC ~0.97)	Tool for triage and referral in ATT‐ALF
Abbara et al. 2017 [47]	UK	Retrospective cohort	Patient on ATT at tertiary center	1529	105	ALT > 3× ULN, or ALP > 2× ULN + bilirubin > 1× ULN, or Bil. > 2× ULN + causality (RUCAM)	53% DILI ≤ 2 weeks 88% DILI ≤ 8 weeks	Clinical, DILI timing and outcomes	Mortality‐5%, Only 1/4th‐DILI patients met ATS/BTS criteria for pre‐emptive monitoring	Early monitoring strategy in all patients irrespective of risk factors
Wang et al. 2020 [89]	China	Retrospective cohort	ATT‐DILI in‐patients in tertiary center	155	155	AST/ALT > 5× ULN, or ALP > 2× ULN or Bil. > 2× ULN, or INR > 1.5× ULN with any AST/ALT/ALP elevation + causality (RUCAM)	Median: 7 weeks (in ALF group) Median: 3–4 weeks (in non‐ALF group)	ALF%, mortality, predictors	ALF‐35%, mortality ~10%. Predictors‐Bilirubin, AST, TLC, platelet, preexisting liver disease	ATT‐ALF has high mortality and early detection is crucial
Zhao et al. 2020 [90]	China	Retrospective cohort	ATT‐DILI hospitalized patients in tertiary center	140	140	Symptoms + abnormal LFT + causality (RUCAM)	Mean (range): 24 days (7–90 days)	Clinical outcomes	Mild to severe liver injury‐79%, ALF‐21%; mortality in ALF‐34%; Overall mortality‐7%; Risk factors for severity‐ Female, combination therapy, rechallenge	Frequent LFT monitoring after rechallenge ATT
Shen and Liu 2025 [91]	China	Retrospective cohort	TB with MASLD on ATT; single‐center	120	28	ALT > 3× ULN; or ALP > 2× ULN	Mean ± SD: 30.4 ± 17.6 days	Incidence, risk factors	DILI‐ 23%; lean MASLD increase ATT‐DILI risk; high‐risk factors‐Low BMI and high FIB‐4	Close monitoring in Lean MASLD particularly with fibrosis

Abbreviations: ALF, acute liver failure; ALP, alkaline phosphatase; ALT, alanine transaminase; AST, aspartate transaminase; ATS, American Thoracic Society; ATT, anti‐tubercular treatment; Bil., bilirubin; BTS, British Thoracic Society; CLD, chronic liver disease; CYP2E1, cytochrome P4502E1; DILI, drug‐induced liver injury; GGT, gamma glutamyl transferase; GST, glutathione S‐transferase gene; HBV, hepatitis B virus; HCV, hepatitis C virus; INR, international normalized ratio; LFT, liver function test; MASLD, metabolic dysfunction‐associated steatotic liver disease; NAC, *N*‐acetylcysteine; NAT2, *N*‐acetyltransferase 2 gene; RUCAM, Roussel Uclaf Causality Assessment Method (structured tool for causality assessment); ULN, upper limit of normal.

### 7.5 Role of *N*‐Acetylcysteine (NAC)

Animal study has shown the protective role of NAC in isoniazid and rifampicin induced DILI. In a human study, 60 new TB patients were randomized into two groups. Group I received standard first‐line ATT, and Group II received the same regimen with NAC (1200 mg/day). ATT‐DILI occurred in 37.5% of patients in Group I and none in Group II. However, the mean duration of the study was very less, and larger studies are needed to reach a conclusion for guiding decisions related to the addition of NAC as a protective therapy [92]. Another RCT explored the role of NAC in treating ATT‐DILI. A total of 102 ATT‐DILI hospitalized patients were randomized to NAC and placebo group. Although NAC significantly reduced length of stay, there was no difference in raised transaminases recovery and mortality [66]. A recent network meta‐analysis found that NAC (1200 mg/day) significantly reduced the occurrence of ATT‐DILI as compared to placebo [70]. However, this was based on a single prior RCT. A small prospective cohort with predominant ATT‐ALF showed significantly lower mortality with NAC as compared to controls (20% vs. 75%) [93]. A systematic review indicated possible advantages of NAC therapy in non‐acetaminophen‐related drug‐induced ALF [94].

Therefore, for prevention of ATT‐DILI, evidence suggests possible benefits but it has not been adopted by any guidelines. In established ATT‐DILI, it is reasonable to start NAC if the patient gets hospitalized, expecting only a shorter stay. In ATT‐ALF, NAC should be started early. Transplant decisions should not be delayed in view of high mortality.

### 7.6 Role of Other Interventions

Besides NAC, various interventions have been used for preventing ATT‐DILI or as an adjunctive treatment. Ursodeoxycholic acid (UDCA) and some herbal formulations may improve cholestatic and mixed‐pattern enzyme abnormalities, but evidence is insufficient [95]. There is no role of silymarin, l‐carnitine, Vit C/D/A, and glutathione [70].

## 8 Recent Advances in ATT‐DILI

Considering the fact that increasing age is the most important predisposing factor for ATT‐DILI, telomere can be a future potential biomarker to predict ATT‐DILI. According to a recent study, longer relative telomere length (RTL) is significantly associated with more susceptibility to ATT‐DILI [96]. Elevation of RTL was also associated with increased levels of liver enzymes in patients with or without ATT‐DILI, suggesting that aberrant telomere length is an important mediator for hepatic injury.

Another study from China reported that combined 5‐hydroxymethylcytosine (5‐hmC) levels of HLA‐B and HLA‐DQB1 as diagnostic biomarkers of ATT‐DILI [97]. This study was based on previous genome‐wide analysis which showed that DNA methylation levels of HLA‐B and HLA‐DQB1 genes were low in individuals with ATT‐DILI compared to those without. Thus, both telomere length and 5‐hmC levels of HLA‐B and HLA‐DQB1 genes can be future promising markers to diagnose ATT‐DILI.

Monitoring of ATT‐DILI still depends on transaminases which are neither always specific to liver nor sensitive to early injury. Emerging biomarkers such as microRNA‐122 (miR‐122) and cytokeratin‐18 (K18) are a liver‐specific microRNA and liver structural protein, respectively. In a study, patients who developed ATT‐DILI had markedly higher miR‐122 levels as compared to ATT tolerant patients, and levels remained unaffected by NAC infusion [86]. This highlights its diagnostic rather than monitoring role. Similarly, cytokeratin‐18 (K18, both total and caspase‐cleaved fragments) has been shown to increase in parallel with ALT during ATT‐DILI in African and European cohorts [98]. Preliminary data show that microRNA‐192 (miR‐192), together with miR‐122, may lead to a multi‐miRNA signature for ATT‐DILI, but results are inconsistent and need confirmation [99].

Beyond these, other mechanistic markers from the broader DILI field such as high‐mobility group box 1 (HMGB1), glutamate dehydrogenase (GLDH), and circulating mitochondrial DNA (mtDNA) are emerging as more liver‐specific biomarkers and give information regarding pathogenic pathways [100].

Future efforts should focus on developing biomarker panels with clear thresholds, validating them in prospective ATT cohorts, and building point‐of‐care assays. Integration with pharmacogenetics and omics data can enable high accuracy risk prediction leading to personalized ATT regimens.

## 9 Conclusion

DILI is unpredictable with ATT due to its idiosyncratic nature. It is important to be aware of its wide spectrum from asymptomatic raised transaminases to ALF because hepatic adaptation does not require any modification or cessation of ATT and early detection of clinically significant DILI by frequent monitoring results in better prognosis. Prompt withdrawal of all the potential hepatotoxic drugs is the key step in the management followed by sequential restart with incremental dosage of least hepatotoxic anti‐tubercular drug first, after raised transaminases settle. More studies are needed for proper positioning of newer anti‐tubercular drugs in modified ATT regimens. Future research is needed to identify specific biomarkers for diagnosing ATT‐DILI.

## Funding

The authors have nothing to report.

## Conflicts of Interest

The authors declare no conflicts of interest.

## Data Availability

The data that support the findings of this study are available from the corresponding author upon reasonable request.
